# Reappraisal of Human HOG and MO3.13 Cell Lines as a Model to Study Oligodendrocyte Functioning

**DOI:** 10.3390/cells8091096

**Published:** 2019-09-17

**Authors:** Kim M. A. De Kleijn, Wieteke A. Zuure, Jolien Peijnenborg, Josje M. Heuvelmans, Gerard J. M. Martens

**Affiliations:** 1 Department of Molecular Animal Physiology, Donders Institute for Brain, Cognition and Behaviour, Centre for Neuroscience, Faculty of Science, Radboud University, 6525AJ Nijmegen, The Netherlands; 2 NeuroDrug Research, 6525 HP Nijmegen, The Netherlands

**Keywords:** cell line, differentiation, HOG, immature oligodendrocyte, Krabbe’s disease, oligodendrocyte, mature oligodendrocyte, MO3.13, myelin, multiple sclerosis, schizophrenia, SH-SY5Y

## Abstract

Myelination of neuronal axons is essential for proper brain functioning and requires mature myelinating oligodendrocytes (myOLs). The human OL cell lines HOG and MO3.13 have been widely used as in vitro models to study OL (dys) functioning. Here we applied a number of protocols aimed at differentiating HOG and MO3.13 cells into myOLs. However, none of the differentiation protocols led to increased expression of terminal OL differentiation or myelin-sheath formation markers. Surprisingly, the applied protocols did cause changes in the expression of markers for early OLs, neurons, astrocytes and Schwann cells. Furthermore, we noticed that mRNA expression levels in HOG and MO3.13 cells may be affected by the density of the cultured cells. Finally, HOG and MO3.13 co-cultured with human neuronal SH-SY5Y cells did not show myelin formation under several pro-OL-differentiation and pro-myelinating conditions. Together, our results illustrate the difficulty of inducing maturation of HOG and MO3.13 cells into myOLs, implying that these oligodendrocytic cell lines may not represent an appropriate model to study the (dys)functioning of human (my)OLs and OL-linked disease mechanisms.

## 1. Introduction

In the central nervous system, axons are ensheathed by myelin which supports neuronal conduction velocity, and provides metabolic and trophic support to neurons [1]. Myelin sheaths are produced by cells of the oligodendrocyte (OL) lineage in which OL precursor cells (OPCs) differentiate via immature OLs (imOLs) and mature OLs (mOLs) into myelinating OLs (myOLs) [1,2,3]. Transcriptome analysis has indicated two sequential stages of in vitro myelination by OLs and that these are characterized by the expression of stage-specific transcription factors and myelin-related proteins [4]. The ‘early-myelination stage’ is specified by the expression of myelin basic protein (MBP), proteolipid protein 1 (PLP1), 2′,3′-Cyclic-nucleotide 3′-phosphodiesterase (CNP) and UDP-galactose ceramide (UGT8A), and the ‘late stage’ by myelin-associated glycoprotein (MAG), myelin-oligodendrocyte basic protein (MOBP), myelin-oligodendrocyte glycoprotein (MOG), myelin and lymphocyte protein (MAL), aspartoacylase (ASPA) and plasmolipin (Tm4sf11) expression [4]. The ‘early-myelination stage’ genes will be referred here to as mOL markers and the ‘late-stage’ genes as myOL markers.

For in vitro functional studies on OLs, the human oligodendrocytic cell lines HOG and MO3.13 are often used. The HOG cell line is directly derived from an oligodendroglioma [5] and the MO3.13 cell line has been constructed by fusion of a rhabdomyosarcoma with a population of primary OLs purified from a mixed human glial population [6]. The HOG and MO3.13 cell lines have been used to study processes involved in OL differentiation and dysfunction [7], immune-mediated injury [8], toxicity of compounds and metals [9], and viral infection [10]. Moreover, these cell lines have been employed as models to examine the cell-biological processes implicated in a number of OL-linked diseases, such as multiple sclerosis [11], Krabbe’s disease [12] and schizophrenia [13]. 

Unfortunately, the protocols used to grow and differentiate HOG and MO3.13 cell lines are largely inconsistent. The HOG cells have been differentiated with N2 supplement (sodium selenite, thyroid hormone T3, insulin, transferrin) [7,8,14,15,16,17], N2 supplement complemented with putrescine, dibutyryl-cAMP (db-cAMP) and 3-isobutyl-1-methylxanthine (IBMX) [10,18,19,20,21,22,23] or N2 supplement complemented with D-biotin and progesterone [24,25,26,27]. Differentiation protocols for MO3.13 cells ranged from the use of phorbol 12-myristate 13-acetate (PMA) [8,12,13,14,28,29,30,31,32,33,34,35,36,37,38,39,40,41,42,43,44,45,46,47,48,49,50,51,52] to serum-deprivation [9,53,54,55,56,57,58,59] and N2 supplement with combinations of progesterone, hydrocortisone, D-biotin and putrescine [7,14,25,34,60]. Furthermore, nearly all (~90%) differentiation studies on HOG and MO3.13 cells reported thus far did not use multiple markers and did not use myOL markers to phenotype the cells. The study that most extensively characterized undifferentiated and differentiated HOG and MO3.13 cells did use multiple OL-related, including myOL, markers, but only one marker for cell types other than OLs and one (HOG) or two (MO3.13) differentiation protocols were tested [14]. Overall, the studies that did perform phenotyping did not lead to a consensus in the expression profiles of OL-related markers in the differentiated HOG and MO3.13 cells. 

From the above, it is clear that in the previously reported differentiation studies on HOG and MO3.13 cells a large variety of protocols was used that has led to an ambiguous situation in the field, including the question as to whether these cells are able to differentiate into a myOL phenotype. In the present study, therefore, we performed a series of differentiation experiments with a number of protocols of previously published studies on cell lines, including the HOG and MO3.13 cell lines [10,30], as well as protocols often used to differentiate primary OLs and stem cells into a myOL phenotype [61,62]. We quantified mRNA and protein expression levels in the undifferentiated and differentiated HOG and MO3.13 cells, not only for multiple OL lineage stage markers, including myOL markers, but also markers for astrocytes, neurons, neural progenitor cells and Schwann cells. Since studies dealing with HOG or MO3.13 cells in the presence of myelin-receiving substrate cells have not yet been reported, we also aimed to stimulate myelin formation in HOG or MO3.13 co-culture experiments with differentiated neuroblastoma-derived neuronal SH-SY5Y cells [63]. As such, we evaluated the validity of the HOG and MO3.13 cell lines as human cell-line-based models to study OL cell biology in vitro.

## 2. Materials and Methods

### 2.1. General Reagents 

DMEM/F12(1:1), antibiotic-antimycotic (AA), fetal bovine serum (FBS) and Glutamax supplement were from Gibco. EMEM and SensiFAST Sybr No-ROX cycling reagent (Bioline, London, UK) were from Lonza. All N2.1, N2.2 and T3 medium components (Table 1), PMA and TRI reagent were from Sigma-Aldrich (St. Louis, MO, USA). The cDNA synthesis kit and Neurobasal medium were purchased from Thermo Fisher. The sources of small molecules and reagents used for co-culture experiments are mentioned in Table S1.

### 2.2. HOG and MO3.13 Cell Culture and Differentiation

The HOG and MO3.13 cells were grown in EMEM with 15% heat-treated FBS (htFBS), 1% glutamax and 1% AA, and passaged twice per week (maximum passage number 35). Mycoplasm tests (MycoAlert Detection Kit, Lonza) were performed tri-annualy to assure the bacteria-free status of cell cultures. Differentiation was induced in HOG and MO3.13 according to the protocols specified in Table 1 at an initial cell plating density of 1.5 × 10^4^ cells/cm^2^. The HOG and MO3.13 cells were differentiated in N2.1, N2.2 or T3 medium for five days, with fresh medium applied after 48 h. The MO3.13 cells were differentiated in PMA-containing medium for seven days, with fresh medium applied every 48 h. The HOG cells were a kind gift of Dr. José Antonio López Guerrero (University of Madrid, Spain) and the MO3.13 cells were a kind gift of Dr. Neil Cashman (University of British Columbia, Vancouver, BC, Canada). The neuroblastoma cell line SH-SY5Y was purchased from ATCC (Manassas, VA, USA).

### 2.3. Co-Culture Experiments

SH-SY5Y cells were cultured and differentiated as described [64]. In brief, cells were plated at a confluency of 5.0 × 10^5^ cells/cm^2^ in a plate coated with Matrigel (1:100; Corning) according to manufacturer’s instructions. For 6 days, differentiation medium (EMEM, 2.5% htFBS and 1% AA, 10 μM all-trans retinoic acid (ATRA)) was added to SH-SY5Y cultures. Cells were then passaged 1:1 onto a new Matrigel-plate with 0.05% Trypsin-EDTA and differentiation medium (EMEM, 1% htFBS and 1% AA 10 µM ATRA) was added. At day 10, the cells were passaged 1:1 with 0.05% Trypsin-EDTA onto a new Matrigel-plate. Final differentiation medium (Neurobasal medium, 20 mM KCl, 20 mM B-27 supplement, 1% AA, 50 ng/mL brain-derived neurotrophic factor (BDNF), 2 mM db-cAMP and 10 µM ATRA) was added every 3 days. Co-cultures of SH-SY5Y and HOG cells or SH-SY5Y and MO3.13 cells were performed with a number of different protocols that were assembled from elements described in Table S1.

### 2.4. Glucose and Serum Deprivation Experiments With HOG and MO3.13

HOG and MO3.13 cells were plated and attached for 24 h in growth medium. Medium was then switched to growth medium without FBS, growth medium without Glutamax supplement, or growth medium without FBS and Glutamax supplement for four days. Medium was refreshed after 48 h. Live cells were imaged with the EVOS FL auto 2 live-cell imaging system (Thermo Fisher Scientific, Waltham, MA, USA) at 37 °C and 5% CO_2_ for microscopy measurements. NucBlue (Thermo Fisher Scientific) was added to each well to stain the nuclei 30 min prior to imaging. Cell count and fluorescence intensity quantification of at least 25 fields per well was performed with Health Profiling toolkit, Cellomics software (Thermo Fisher Scientific).

### 2.5. RNA Isolation and cDNA Synthesis

For RNA isolation, cells were lysed with TRI reagent and chloroform was used for phase separation. The RNA was incubated with glycogen and precipitated with isopropanol. The RNA pellet was washed twice in ice-cold 75% ethanol by centrifugation. Air-dried RNA was resuspended in nuclease-free water. The RNA concentration and purity were measured with a DeNovix spectrophotometer. To test RNA quality, 1% agarose gel electrophoresis was performed to confirm 18S/28S band integrity. Total RNA samples were treated with DNAse and ethylenediaminetetraacetic acid (EDTA) was added to protect RNA during inactivation of DNAse. The cDNA synthesis was performed with a master mix containing 5 × FSB, 40 U/µL RNAse inhibitors, 200 U/µL RevertAid H minus Reverse Transcriptase enzyme, MilliQ, 0.2 µg/µL random hexamer primer, 10 mM dNTPs (each).

### 2.6. Real-Time Quantitative Polymerase Chain Reaction (RT-qPCR)

Real-time quantitative polymerase chain reaction (RT-qPCR) was performed with a mastermix containing the forward (FW) primer, reverse (RV) primer, MilliQ water and SensiFAST Sybr No-ROX cycling reagent (for primer sequences, see Table S3). The RT-qPCR reaction was performed with cDNA diluted 1:30 in the mastermix in a thermo-optical analyser (Corbett Rotor-Gene 6000). Melt-curve analysis was performed to confirm correct amplicon size. Primers for RT-qPCR were designed with PrimerBlast and primers were validated with human motor cortex cNDA (obtained from Netherlands Brain Bank, Amsterdam and cDNA prepared as described above) as positive control before use. In each experiment, four housekeeping genes (*GAPDH*, *EIF4A2*, *YWHAZ*, *PPIA*) were run. Normalization of gene of interest values was performed according to [65], with a normalization factor based on two housekeeping genes with low M-value calculated by GeNorm. The level of mRNA expression (Q-value) for each sample was calculated by:

(1) $$Q-\mathrm{value}\;=\;{\left(\mu \;\mathrm{amplification}\;\mathrm{efficiency}\right)}^{\left(\min.\;\mathrm{take}\;\mathrm{off}–\mathrm{sample}\;\mathrm{take}\;\mathrm{off}\right)}$$

This formula takes into account the mean amplification efficiency for one primer pair and scales all values between 0 and 1 for relative comparisons [65].

### 2.7. Immunocytochemistry

For immunocytochemistry, cells were fixed in 2 or 4% paraformaldehyde for 15 min at room temperature, washed twice with PBS1X and incubated for one hour at RT with blocking buffer (BB+; 5% goat/donkey/horse serum, 1% BSA, 1% Glycine, 0.1% d-lysine, 0.4% Triton-X in PBS1X) or with BlockHen blocking solution (Aves Labs, Davis, CA, USA). Antibodies purchased from Aves Labs (Table S2) were incubated overnight at 4 °C in PBS-T 0.1%, while the other antibodies (Table S2) were incubated overnight at 4 °C in BB+. After two PBS1X washes, cells were incubated with secondary antibodies (Thermo Fisher/Aves Labs) and DAPI nuclear stain (Sigma) for two hours at RT. Aves labs anti-chicken antibodies were diluted in PBS-T 0.1%, other secondary antibodies in BB+. FluoroMyelin Red (FLMred) staining on 4% PFA-fixed cells was performed overnight at a 1:100 dilution in PBS, while for live-cell imaging FLMred was diluted 1:100 in medium and incubated overnight. Live cells were stained with O1 and O4 antibodies diluted in medium, and subsequently fixed with 4% PFA followed by detection with secondary antibodies. Immunocytochemistry for L1CAM and Jagged1 was performed on cells blocked in BB+ without Triton-X and antibodies were diluted in PBS1X. For all experiments, imaging was performed in the EVOS FL auto 2 live-cell imaging system (Thermo Fisher Scientific) and images were taken with a 20X long-working distance EVOS2 objective. A minimum of 25 fields was used per well for all analyses. To analyse neurite calibers in differentiated SH-SY5Y cells, 2000 cell bodies per well were analysed with the Neurite Detection toolkit, HCS Studio Cell Analysis software (Thermo Fisher Scientific).

### 2.8. Western Blot Analysis

For Western blot analysis, cultured HOG and MO3.13 cells were collected by scraping and homogenized in sample buffer (62.5 mM Tris-HCl pH 6.8, 2% SDS, 10% Glycerol, 0.01% Bromophenol Blue, β-Mercaptoethanol). Samples were sonified 6 times for 10 s each with 10-second intervals. As a control, human motor cortex (obtained from Netherlands Brain Bank, Amsterdam) was grinded and homogenized in RIPA buffer (Tris-Hcl pH 8.0 50 mM, 150 mM NaCl, 1% NP-40, 0.1% SDS, 0.5% Na-deoxycholate, and protease inhibitors (Roche, Rotkreuz, Switzerland) in a glass potter. Samples were then sonified 10 times 10 s with 10 second breaks and diluted in sample buffer. Protein quantification was performed with BCA assay (Thermo Fisher). Samples were boiled for 5 min at 95 °C, per sample about 10 μg protein was loaded on 12.5% or 8% acrylamide gels, and electrophoresis was started at 70 V for 15 min and then continued at 150 V for 1 h in Tris-Glycine buffer with 0.1% SDS. Blotting onto Amersham Protran 0.45 μm nitrocellulose membranes was performed in Tris-HCl, 20% MeOH, 0.15% SDS for 1 h at 100V and successful transfer was confirmed by Ponceaus dye. Blots were blocked for 1 h by shaking in 5% milk powder (Elk) in PBS. Primary antibodies (Table S2) diluted in 5% milk in PBS-T 0.1% were incubated overnight on a rotator at 4 °C and secondary horseradish peroxidase antibodies (Nordic Immunology) were incubated for 1.5 hour at room temperature on a rotator. Western blotting for MBP and CNPase anti-chicken antibodies was performed without milk, by blocking in PBS-T 0.1% with BlockHen buffer (Aves labs) diluted 1:40 in PBS-T 0.1% for 1 h at RT and all primary and secondary antibodies were diluted in PBS-T 0.1%. Detection of protein signals was performed with LumiLight Western blotting substrate (Roche) in a LAS4000 Quant detection system (GE Healthcare) and signals were analysed in ImageJ.

### 2.9. Statistical Analysis

Statistical analysis was performed in IBM SPSS Statistics (Version 24). All effects were tested at a confidence level of 5%. Sample variation was calculated as the standard error of the mean (SEM), unless otherwise indicated.

## 3. Results

### 3.1. Differentiation of HOG Cells Does Not Induce a MyOL Expression Profile

**Figure 1:**
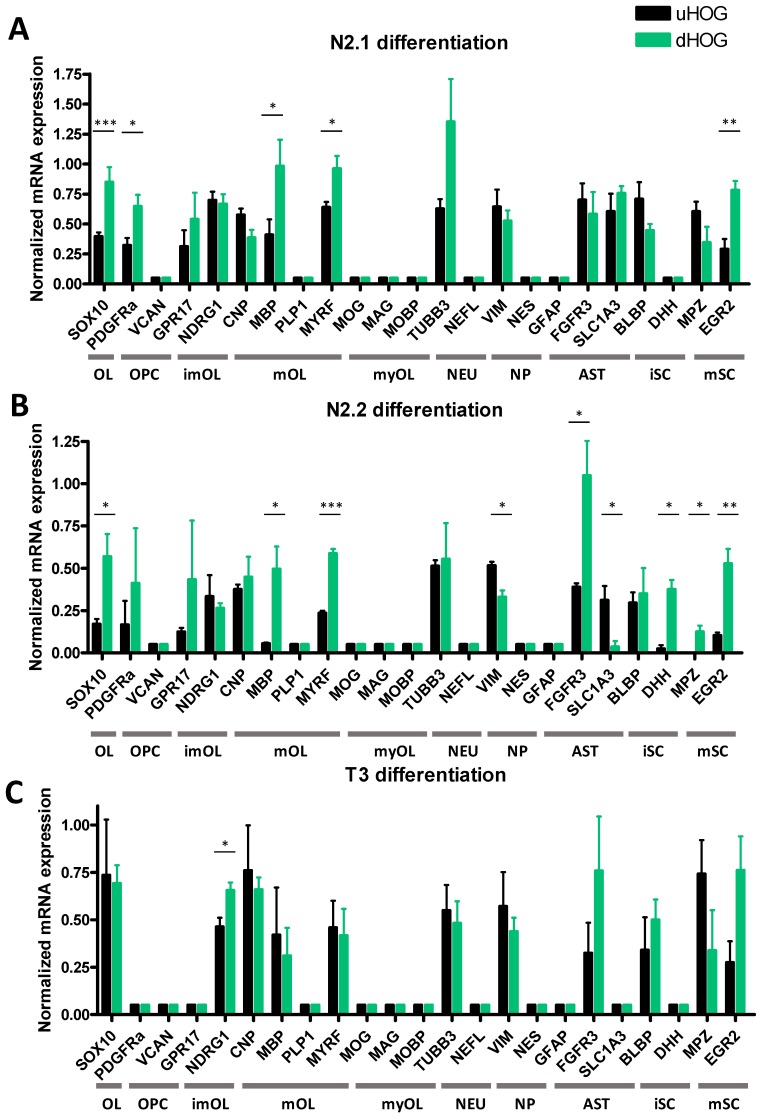
Normalized mRNA expression in undifferentiated HOG cells (uHOG) and HOG cells following differentiation (dHOG) with (**A**) N2.1 medium, (**B**) N2.2 medium or (**C**) T3 medium. OL: oligodendrocyte lineage, OPC: oligodendrocyte precursor, imOL: immature oligodendrocyte, mOL: mature oligodendrocyte, myOL: myelinating oligodendrocyte, NEU: neuronal, NP: neural progenitor, AST: astrocyte, iSC: immature Schwann cell, mSC: mature Schwann cell. Independent samples *t*-tests are based on triplicates in three independent experiments (*n* = 3). *p*-values: * < 0.05, ** < 0.01, *** < 0.001.

We differentiated HOG cells for five days in (1) N2.1 differentiation medium, (2) N2.2 differentiation medium and (3) T3 differentiation medium (for medium composition, see Table 1). The three differentiation protocols did not produce extensive morphological changes in the HOG cells (Figure S1B–D). Following differentiation of HOG cells with N2.1 medium, mRNA expression of OL lineage marker SOX10 (t(6) = 7.1368, *p* = 0.0004), OPC marker PDGFRα (t(6) = 3.0133, *p* = 0.0296), of mOL markers MBP (t(6) = 3.5476, *p* = 0.0121) and MYRF (t(6) = 2.7667, *p* = 0.05), and of mature Schwann cell marker EGR2 (t(6) = 4.2336, *p* = 0.0055) was increased (Figure 1A). HOG cells differentiated with N2.2 medium also showed an increased mRNA expression of SOX10 (t(4) = 2.9292, *p* = 0.0428), MBP (t(4) = 3.3161, *p* = 0.0295) and MYRF (t(4) = 11.1217, *p* = 0.00044), and a decrease in mRNA expression of neural progenitor marker VIM (t(4) = 4.0776, *p* = 0.0151) and astrocyte marker SLC1A3 (t(4) = 3.1769, *p* = 0.0336), while mRNA expression of another astrocyte marker (FGFR3) was increased (t(4) = 3.1769, *p* = 0.0336) compared to undifferentiated cells (Figure 1B). Furthermore, mRNA expression of immature Schwann cell marker DHH (t(4) = 4.5408, *p* = 0.0200) and mature Schwann cell marker for MPZ (t(4) = 3.3559, *p* = 0.0284) and EGR2 (t(4) = 4.7551, *p* = 0.0089) was increased in the N2.2-differentiated HOG cells (Figure 1B). Differentiation of HOG cells with T3 increased mRNA expression of only the imOL marker NDRG1 (t(4) = 2.9903, *p* = 0.0403) (Figure 1C). In none of the conditions (undifferentiated, and N2.1-, N2.2- and T3-differentiated HOG cells), mRNA expression of the early OPC marker VCAN was found (Figure 1A–C), while the astrocyte marker FGFR3 was expressed in undifferentiated HOG cells and still expressed after differentiation (Figure 1A–C). Moreover, in none of the HOG experiments we found mRNA expression of mOL marker PLP1, myOL markers MOG, MOBP and MAG, astrocyte marker glial fibrillary acidic protein (GFAP) and neuronal marker neurofilament light chain (NEFL) in undifferentiated or differentiated cells (Figure 1A–C).

**Figure 2:**
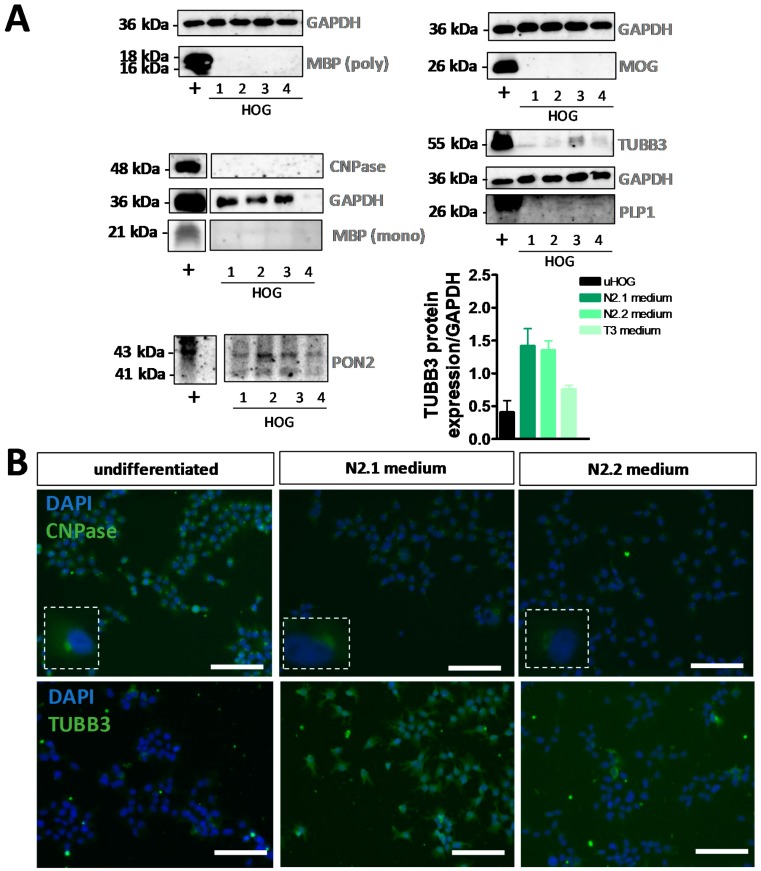
Analysis of protein expression in undifferentiated and differentiated HOG cells. (**A**) Western blot analyses of myelin basic protein (MBP) (polyclonal), myelin-oligodendrocyte glycoprotein (MOG), CNPase, MBP (monoclonal), class III β-tubulin (TUBB3), proteolipid protein 1 (PLP1) and paraoxonase 2 (PON2) in (+) human motor cortex, (1) undifferentiated HOG cells, (2) differentiated HOG cells following incubation in N2.1 medium, (3) N2.2 medium or (4) T3 medium (normalized to *GAPDH* expression). Western blot analysis was performed for two independent experiments (*n* = 2). (**B**) Example images of immunocytochemistry for CNPase and TUBB3 in undifferentiated HOG cells and HOG cells differentiated with N2.1 medium or N2.2 medium. Scale bar = 50 µm.

The increase of MBP mRNA expression in N2.1- and N2.2-differentiated HOG cells was not confirmed at the protein level, since no MBP protein was detected in (un)differentiated HOG cells with immunocytochemistry (Figure S3A) or Western blot analysis (Figure 2A) using polyclonal as well as monoclonal antibodies. A discrepancy in mRNA and protein expression was also observed for CNP (Figure 1A–C, Figure S3A and Figure 2A,B), whereby the observed perinuclear immunocytochemical signal was probably due to nonspecific antibody binding. The trend towards increased TUBB3 mRNA expression after N2.1 incubation (Figure 1A) was confirmed at the protein level by both Western blot analysis (Figure 2A) and immunocytochemistry (Figure 2B and Figure S3A). Furthermore, immunocytochemistry showed an absence of surface expression of the mOL markers sulfatide (O4) and galactosylceramide (O1 (ex)) on living HOG cells, while protein expression of astrocyte markers GFAP and PON2 (PON2; Figure 2A), neuronal markers NSE and TUBB3, and neural progenitor markers VIM and NES was found both in undifferentiated, and N2.1- and N2.2-differentiated HOG cells (Figure S3A,C). The absence of PLP1 and MOG expression at the protein level was shown by Western blot analysis (Figure 2A) and immunocytochemistry (Figure S3A).

The lack of expression of the myOL markers in HOG cells was likely not due to cellular stress since the absence of glutamine and/or serum in the differentiation medium for four days did not result in nuclear to cytoplasmic translocation of the stress-granule protein HuR (Figure S1A). Glutamine deprivation (*p* = 0.002), and glutamine and serum deprivation (*p* = 0.000) decreased only the cell proliferation rate of HOG cells (Figure S1A). Taken together, our HOG studies show that the three differentiation protocols applied here did not generate a myelinating phenotype. 

### 3.2. Differentiation of MO3.13 Cells Does Not Induce a MyOL Expression Profile

**Figure 3:**
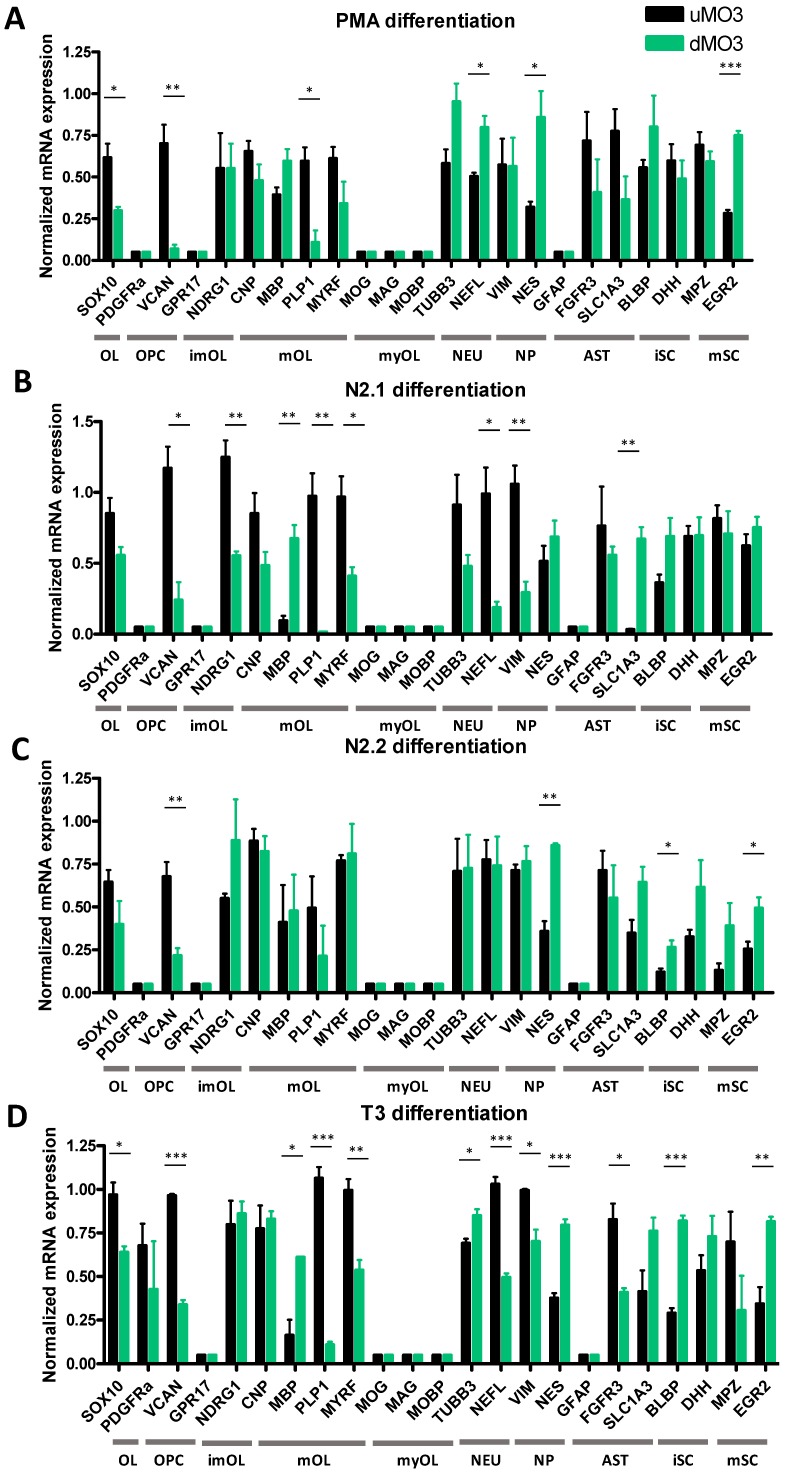
Normalized mRNA expression in undifferentiated MO3.13 cells (uMO3) and MO3.13 cells following differentiation (dMO3) with (**A**) PMA, (**B**) N2.1 medium, (**C**) N2.2 medium or (**D**) T3 medium. OL: oligodendrocyte lineage, OPC: oligodendrocyte precursor, imOL: immature oligodendrocyte, mOL: mature oligodendrocyte, myOL; myelinating oligodendrocyte, NEU: neuronal, NP: neural progenitor, AST: astrocyte, NP: neural progenitor, iSC: immature Schwann cell, mSC: mature Schwann cell. Independent samples *t*-tests are based on triplicates in three independent experiments (*n* = 3). *p*-values: * < 0.05, ** < 0.01, *** < 0.001.

MO3.13 cells were differentiated for five days in (1) N2.1 medium, (2) N2.2 medium and (3) T3 medium or for seven days in (1) PMA medium (Table 1). Differentiation of MO3.13 cells with N2.1 medium generated a heterogeneous pool of multipolar, bipolar and non-arborized cell phenotypes, while PMA medium mainly induced spindle-like bipolar cells. The morphological changes after differentiation with N2.2 or T3 medium were less apparent (Figure S2B–E). Following treatment with PMA, differentiated MO3.13 cells showed decreased mRNA expression of SOX10 (t(4) = 3.5915, *p* = 0.0299), OPC marker VCAN (t(4) = 5.4596, *p* = 0.0055) and mOL marker PLP1 (t(4) = 4.4364, *p* = 0.0114), while mRNA expression of neuronal marker NEFL (t(4) = 4.0704, *p* = 0.0152), neural progenitor marker NES (t(4) = 3.3631, *p* = 0.0282) and Schwann cell marker EGR2 (t(4) = 13.4521, *p* = 0.0002) was increased (Figure 3A). In MO3.13 cells differentiated with N2.1 medium, mRNA expression of VCAN (t(4) = 4.7086, *p* = 0.0181), imOL marker NDRG1 (t(4) = 5.6992, *p* = 0.0047), and mOL markers MYRF (t(4) = 3.4955, *p* = 0.0250) and PLP1 (t(4) = 5.9266, *p* = 0.0041) was decreased, while mRNA expression of MBP (t(4) = 5.7572, *p* = 0.0045) and astrocyte marker SLC1A3 (t(4) = 7.6041, *p* = 0.0016) was increased. Decreased mRNA expression of neuronal marker NEFL (t(4) = 4.2208, *p* = 0.0135) and neural progenitor marker VIM (t(4) = 5.0691, *p* = 0.0071) was found in N2.1-differentiated MO3.13 cells (Figure 3B). Differentiation with N2.2 medium decreased mRNA expression of VCAN (t(4) = 4.8078, *p* = 0.0086) and increased expression of NES (t(4) = 8.2606, *p* = 0.0012) and (im)mature Schwann cell markers BLBP (t(4) = 3.1857, *p* = 0.0334) and EGR2 (t(4) = 3.1630, *p* = 0.0341) (Figure 3C). MO3.13 cells differentiated with T3 showed decreased mRNA expression of SOX10 (t(4) = 4.1794, *p* = 0.0139), VCAN (t(4) = 22.1698, *p* = 0.0001), MYRF (t(4) = 5.2495, *p* = 0.0063) and PLP1 (t(4) = 14.4456, *p* = 0.0001), while mRNA expression of MBP (t(2) = 5.0871, *p* = 0.0365), neuronal marker TUBB3 (t(4) = 3.4946, *p* = 0.025) and NES (t(4) = 9.7978, *p* = 0.0006 was increased) (Figure 3D). Expression of NEFL (t(4) = 11.4286, *p* = 0.0003), neural progenitor marker VIM (t(4) = 4.3088, *p* = 0.016), and FGFR3 (t(4) = 4.4012, *p* = 0.0117) decreased and Schwann cells markers BLBP (t(4) = 12.5417, *p* = 0.0002) and EGR2 (t(4) = 4.7178, *p* = 0.0092) increased in T3-differentiated MO3.13 cells (Figure 3D). The early OPC marker GPR17 was not expressed at the mRNA level in undifferentiated and PMA-, N2.1-, N2.2- and T3-differentiated MO3.13 cells, while mRNA expression of the astrocyte markers SLC1A3 and FGFR3 was found both before and after differentiation (Figure 3A–D). Also analogous to the HOG cell studies, in none of the MO3.13 differentiation experiments we found mRNA expression of myOL markers MOG, MOBP and MAG (Figure 3A–D).

**Figure 4:**
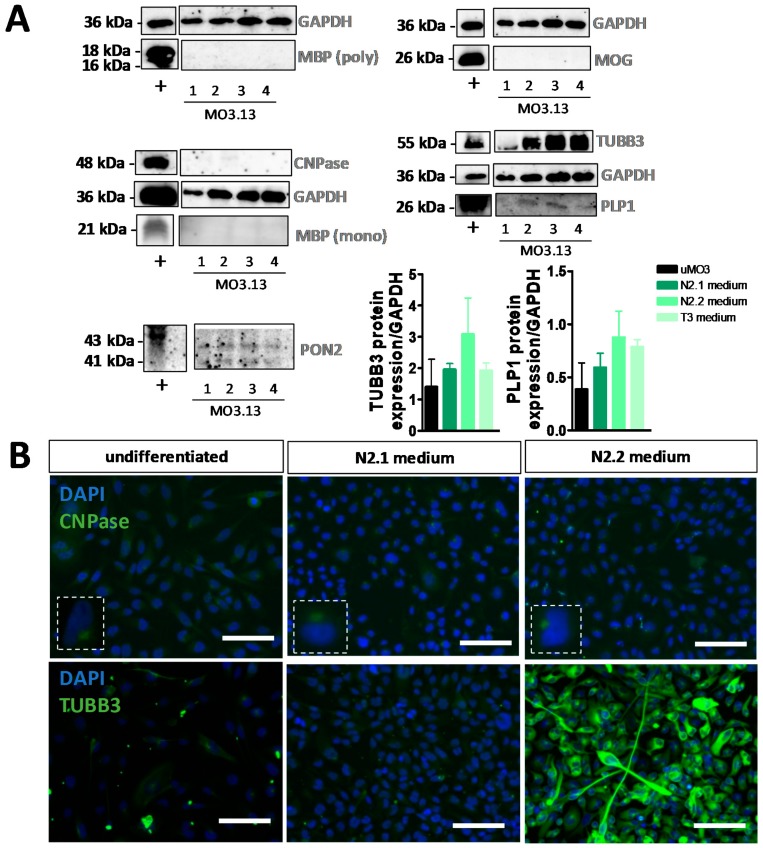
Analysis of protein expression in undifferentiated and differentiated MO3.13 cells. (**A**) Western blot analyses of MBP (polyclonal), MOG, CNPase, MBP (monoclonal), TUBB3, PLP1 and PON2 in (+) human motor cortex, (1) undifferentiated MO3.13 cells, (2) differentiated MO3.13 cells following incubation in N2.1 medium, (3) N2.2 medium or (4) T3 medium (normalized to GAPDH expression). Western blot analyses were performed for two independent experiments (*n* = 2). (**B**) Example images of immunocytochemistry for CNPase and TUBB3 in undifferentiated MO3.13 cells and MO3.13 cells differentiated with N2.1 medium or N2.2 medium. Scale bar = 50 µm.

As with HOG cells, the increase of MBP in N2.1- and T3-differentiated MO3.13 cells at the mRNA level was not confirmed at the protein level, since no MBP was detected in (un)differentiated MO3.13 cells with immunocytochemistry (Figure S3B) or Western blot analysis (Figure 4A) with both polyclonal and monoclonal antibodies. Likewise and also similar to the results obtained with the HOG cells, a discrepancy in the findings for CNP mRNA and protein expression was observed in the (un)differentiated MO3.13 cells (Figure 3B,C, Figure S3B and Figure 4A,B). Furthermore, low but modulated PLP1 protein levels were detected in differentiated MO3.13 cells on Western blots (Figure 4A) and by immunocytochemical analysis of (un)differentiated MO3.13 cells (Figure S3B,D). Immunocytochemical analysis showed also the absence of sulfatide (O4) and galactosylceramide (O1 (ex)) surface expression on living (un)differentiated MO3.13 cells, while astrocyte markers GFAP and PON2 (PON2; Figure 4A), neuronal markers NSE and TUBB3, and neural progenitor markers VIM and NES were expressed both in undifferentiated and N2.1- and N2.2-differentiated MO3.13 cells (Figure S3B,D). The absence of MOG mRNA expression in undifferentiated and differentiated MO3.13 cells is in line with the absence of MOG protein as shown by both Western blot (Figure 4A) and immunocytochemical (Figure S3A) analysis. Note that, due to the small cytoplasmic area of the PMA-differentiated, spindle-like-stretched MO3.13 cells (Figure S2F), only low amounts of protein were recovered from these cells and therefore protein detection on Western blots was hampered.

Again, the absence of myOL marker expression in differentiated MO3.13 was not due to cellular stress caused by the serum and glutamine deprivation in the differentiation medium, since the deprivation only induced a decrease in MO3.13 proliferation rate (*p* = 0.000 for both), but no translocation of stress-granule protein HuR in our cultures (Figure S2A). Thus, none of the four differentiation protocols induced a myelinating phenotype in the MO3.13 cells.

### 3.3. Cell Density Affects mRNA Expression in HOG and MO3.13 Cells 

**Figure 5:**
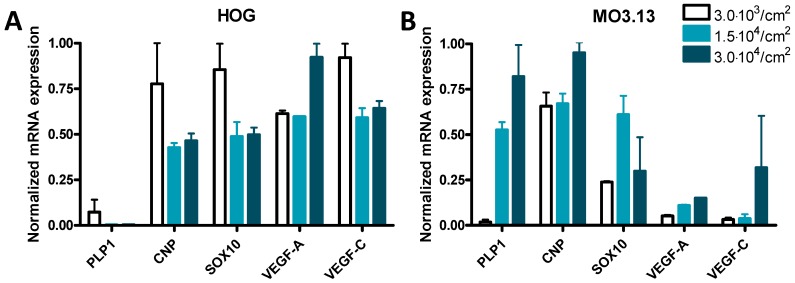
Normalized mRNA expression of PLP1, CNP, SOX10, vascular endothelial growth factor A (VEGF-A) and VEGF-C at various cell densities in (**A**) HOG cells and (**B**) MO3.13 cells. Standard error of the mean (SEM) is based on two technical replicates of one experiment (*n* = 1).

Since the seeding density of cultured cells may affect the experimental outcome [66], and the majority of HOG and MO3.13 studies did not report the cell densities used, we performed mRNA expression studies on undifferentiated cells grown at three different cell densities. While only minor or no expression of PLP1 mRNA was found in HOG cells (Figure 5A), the expression of PLP1 mRNA in MO3.13 cells increased 29-fold and 45-fold at a density of 1.5 × 10^4^ cells/cm^2^ and 3.0 × 10^4^ cells/cm^2^, respectively, when compared to MO3.13 cells grown at 3.0 × 10^3^ cells/cm^2^ (Figure 5B). The mRNA expression of vascular endothelial growth factor C (VEGF-C), VEGF-A, CNP and SOX10 only changed about 1.5-fold by HOG cell density (Figure 5A). The expression level of SOX10 mRNA was about two-fold higher in MO3.13 cells grown at 1.5 × 10^4^ cells/cm^2^ compared to 3.0 × 10^3^ cells/cm^2^, while mRNA expression of CNP, VEGF-A and VEGF-C was virtually not modulated by MO3.13 cell density (Figure 5B).

### 3.4. Co-Cultures of HOG or MO3.13 Cells with Neuronal SH-SY5Y Cells Do Not Display Myelin Formation

**Figure 6:**
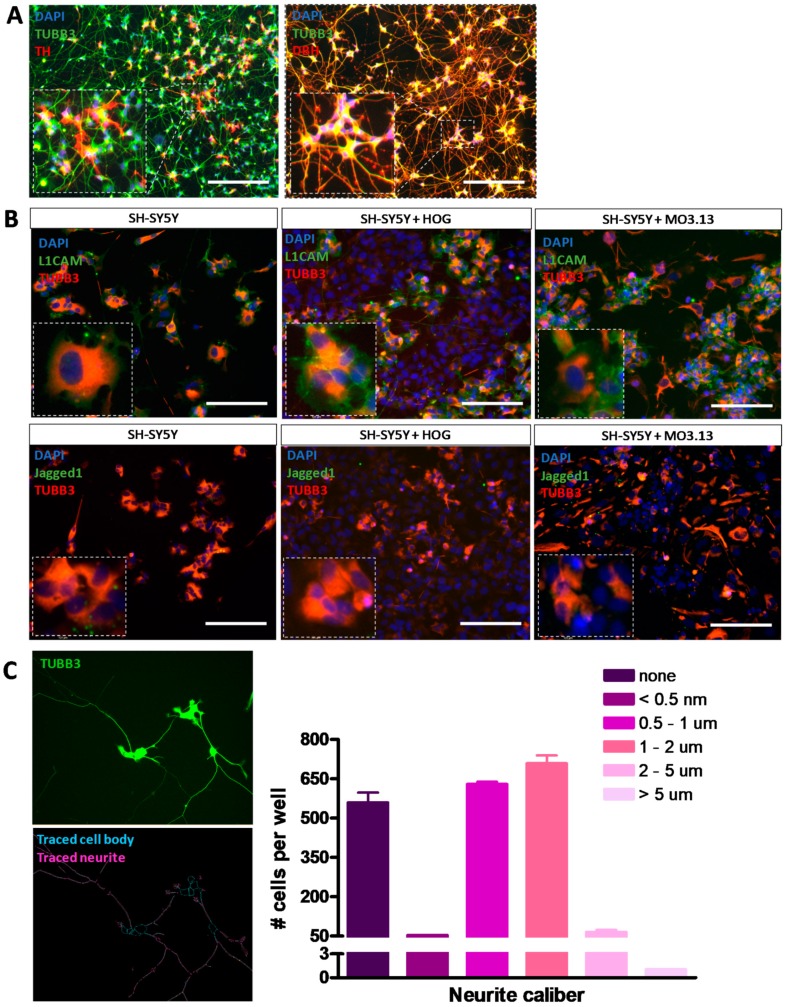
Immunocytochemical analysis of neuronal differentiation in co-cultures of HOG or MO3.13 cells with differentiated neuronal SH-SY5Y cells. (**A**) Representative images of for expression of mature neuronal markers TUBB3 and tyrosine hydroxylase (TH) or dopamine beta-hydroxylase (DBH) in differentiated SH-SY5Y monocultures. (**B**) Representative images of Jagged1 and L1CAM surface expression and TUBB3 signals in SH-SY5Y monoculture, and SH-SY5Y + HOG and SH-SY5Y + MO3.13 co-cultures. Scale bar = 50 µm. (**C**) Quantifications of neurite caliber in low density neurite networks of differentiated SH-SY5Y cells (*n* = 2).

For co-culture experiments, neuronal SH-SY5Y cells were first differentiated using ECM-matrix Matrigel and differentiation factors db-cAMP, BDNF and all-trans retinoic acid [64,67], producing cells with extensive neurite outgrowths (Figure 6A). Immunocytochemical co-localization of TUBB3 with neuronal subtype markers tyrosine hydroxylase (TH) and dopamine beta-hydroxylase (DBH) confirmed the differentiated phenotype (Figure 6A). Moreover, in co-cultures of HOG or MO3.13 cells with differentiated SH-SY5Y cells we observed surface membrane expression of myelin-attractive factor L1CAM, but only low cell surface expression of myelin-repulsive factor Jagged1 in the differentiated SH-SY5Y cells (Figure 6B). Quantification of the neurite caliber in low-density differentiated SH-SY5Y cells showed that the majority of the neurite extensions displayed a caliber of 0.5–2 μm (Figure 6C). 

**Figure 7:**
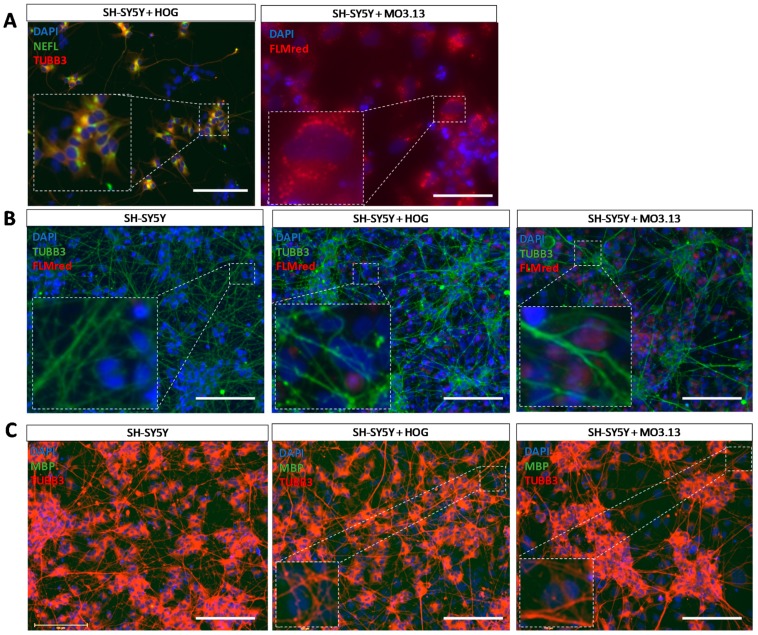
Immunocytochemical analysis of co-cultures of HOG or MO3.13 cells with differentiated neuronal SH-SY5Y cells. (**A**) Representative image of neuronal marker neurofilament light chain (NEFL) and TUBB3 stainings in SH-SY5Y + HOG co-culture and FluoroMyelin Red (FLMred) cytoplasmic lipid droplets in living SH-SY5Y + MO3.13 co-cultures. (**B**) Representative images of TUBB3- and FLMred signals in fixed SH-SY5Y monoculture, and SH-SY5Y + HOG co-culture and SH-SY5Y + MO3.13 co-culture. These images serve as examples for the absence of FLMred overlap with the extensive neurite outgrowth (TUBB3) in our cultures. (**C**) Representative images of the absence of MBP-signals (polyclonal antibody) and the presence of TUBB3-signals in SH-SY5Y monocultures, and SH-SY5Y + HOG and SH-SY5Y + MO3.13 co-cultures. Scale bar = 50 µm.

Co-culture experiments of differentiated SH-SY5Y with HOG or MO3.13 aimed at the stimulation of myelin formation were performed using multiple time periods of co-culturing, time-points of OL addition, OL differentiation protocols, pro-myelinating compounds, pro-myelin growth factors and proteins, and several total cell densities as well as multiple HOG, MO3.13 and SHSY5Y cell ratios (see Table S1). In co-cultures, HOG cells were distinguished from SH-SY5Y cells by a lower expression of TUBB3 and an absence of NEFL (Figure 7A). Since MO3.13 cells express NEFL (Figure 3A–D), these cells could be distinguished in co-cultures by a high cytoplasmic lipid count (Figure 7A), in line with oligodendrocyte lineage cells which show high lipid metabolism in vivo [68]. To detect myelin formation in our cultures, we used the well-established lipid dye FLMred [69]. In none of the conditions used in the SH-SY5Y with HOG or MO3.13 co-cultures, we detected a signal of the specific fluorescent myelin marker FLMred that overlapped with TUBB3-positive neuronal outgrowths, indicating that no myOLs were formed in any of the pro-myelination environments we applied (Figure 7B). Furthermore, under basal conditions (without specific pro-myelinating stimulation), HOG and MO3.13 cells did not mature to MBP-expressing cells in our co-cultures (Figure 7C).

## 4. Discussion

Due to their role in axon ensheathment and broader function in glial-neuron communication, myOLs are essential for proper functioning of neuronal networks [70]. To explore the (dys)functioning of myOLs, the long-established HOG and MO3.13 cell lines have been extensively used as cell models, but the validity of these cell lines has not been fully tested. Studies using HOG and MO3.13 cells often do not report a thorough validation of the (un)differentiated cellular phenotype, since mostly only a single OL or imOL differentiation marker was used (see Table S4 for an overview of previously performed HOG and MO3.13 studies). To investigate the capacity of HOG and MO3.13 cells to differentiate into a myelinating phenotype, we used markers for multiple cell types to perform mRNA and protein expression studies on undifferentiated and differentiated HOG and MO3.13 cells, and co-culture experiments in which neuronal SH-SY5Y cells were aimed to act as tentative myelin substrates.

The process of OL differentiation and maturation towards myelinating cells is amongst others characterized by increases in cell body size, and the length and number of arborisations [71]. Apart from small increases in arborisation length and number, the HOG cells did not display major morphological changes following differentiation with the three protocols used here (N2.1 medium, N2.2 medium and T3 medium). Furthermore, the three differentiation protocols induced mRNA expression of the mOL marker MBP in HOG cells, albeit at low levels, while MBP protein expression was not detected in the differentiated cells by Western blot analysis and immunocytochemistry using both a polyclonal and a monoclonal antibody. Low levels of MBP mRNA have also been previously reported in differentiated HOG cells [14], but at the immunocytochemical level one did [14] and another study did not [24] report MBP protein expression in the differentiated cells. The reason for the MBP mRNA-protein discrepancies is at present unclear, but is not related to the occurrence of MBP pre-mRNA splice variants [72], since our primers for MBP mRNA expression analysis were designed against a region common to the various splice variants. Likewise, discrepancies in findings exist with respect to CNP in that we and others [14] found CNP expression at the mRNA level in undifferentiated HOG cells that was not increased following N2.1 and N2.2 differentiation, while protein expression results varied from decreased [14] and no (our present study) to increased [24] CNPase expression in differentiated HOG cells. Regarding mOL marker PLP1, we also found no induction of mRNA expression and essentially no PLP1 protein on both Western blots and using immunocytochemistry, whereas Bello-Morales, et al. [18,20,21] have reported PLP1 protein expression in differentiated HOG cells, but again with the use of only immunocytochemical analysis. In line with an absence of MBP, CNPase and PLP1 protein expression, none of our differentiation protocols induced HOG expression of the myOL markers MOG, MOBP and MAG, which is observed in terminally differentiated OLs [4]. Remarkably, a previous microarray study has shown increased mRNA expression of these myOL markers in differentiated HOG cells [14], but these microarray data were not validated and the study did not include protein expression analysis of these markers. Taken together, we conclude that the protocols employed in our study presumably differentiated HOG cells towards imOLs, but not myOLs.

In contrast to the HOG cells, the morphology of the MO3.13 cells was clearly changed by two of the four differentiation protocols we used. Differentiation of MO3.13 with PMA yielded bipolar cells with a small cytoplasmic area, reminiscent of Schwann or astrocytic cells, while the N2.1 differentiation protocol produced a heterogeneous set of morphologies, including bipolar and multipolar cells. The four differentiation protocols applied here all increased MBP mRNA levels, which is in line with other reports [14,35,43], but we did not find MBP expression at the protein level, in agreement with other [32,43,60], but not all [40,47,50] studies on differentiated MO3.13 cells. Again, the issue of MBP mRNA-protein expression differences is not related to alternative splicing events, but remains otherwise unclear. We detected CNP mRNA expression in both undifferentiated and differentiated MO3.13 cells, in line with Buntinx et al. [14], while in contrast to another study [34] we did not detect CNPase protein expression in (un)differentiated MO3.13 cells. The various differentiation protocols further caused clearly reduced PLP1 mRNA expression, while we and others [12,34] observed a slight increase at the protein level. Possibly, the effect of cell density on PLP1 mRNA expression (see below) may have contributed to this unexpected result. Finally, an important finding was that, apart from MBP, mRNA and protein expression of the other mOL markers (PLP1, CNP, MYRF) was not upregulated and, in contrast to the previous, not-validated microarray study [14], no expression of myOL markers MOBP, MOG and MAG was found in the differentiated MO3.13 cells. On the basis of these results, we conclude that following application of the four differentiation protocols MO3.13 cells remain in an imOL state and do not differentiate into myOLs.

The conclusion that our protocols did not lead to the differentiation of HOG and MO3.13 cells into myOLs is in line with the results of our co-culture experiments with the neuronal cell line SH-SY5Y that were aimed at stimulating HOG and MO3.13 cells to produce myelin. Still, co-culturing HOG or MO3.13 cells with differentiated SH-SY5Y cells did not result in MBP protein expression, and thus did not induce a myOL phenotype. Of note, a co-culture model based on human cell lines would greatly facilitate high-throughput studies on neuron-glia interactions and drug screening. To our knowledge, only one study co-cultured HOG cells (with CD4+ immune T-cells, [17]) and only one co-cultured MO3.13 cells (with pheochromocytoma-derived PC12 cells, [73]), but neither investigated processes related to myelination. Our HOG/MO3.13 co-culture experiments with SH-SY5Y further involved the application of a wide variety of protocols aimed at stimulating myelination, which included the pro-myelinating compounds Miconazole and Clobetasol [74], modulators of the OL-differentiation-related G-protein-coupled receptors GPR37 and GPR17 (Montelukast, MDL29,951 and Prosaptide, [75,76]), and known OL differentiation factors such as insulin-growth factor-1 (IGF-1), T3 and BDNF [77,78,79]. Moreover, we attempted to induce myelination by increasing SH-SY5Y neuronal activity (e.g. through the use of BrainPhys medium [80]) and by inducing neuronal action potentials relevant to OL-neuron interactions (with ATP, [81]). None of the co-culture conditions led to the induction of myelin formation as evaluated by a myelin-related lipid dye, endorsing the notion that it is difficult to differentiate HOG and MO3.13 into myOLs, even in a co-culture with differentiated SH-SY5Y cells. Interestingly, primary rodent OLs and OLs derived from human induced pluripotent stem cells (iPSCs) are able to form in vitro ‘lamelliform’ membranes enriched in myelin proteins and lipids in the absence of neurons [71,82].

Our differentiated SH-SY5Y cells showed protein expression of the dopaminergic neuronal marker TH, the noradrenergic neuronal marker DBH, the myelin-attractive factor L1CAM and the mature neuronal marker NEFL, which are all related to axonal and dendritic branching and maintenance in neuronal cells [83,84,85,86], and displayed neurite calibers that in principle would allow for myelination [87]. Moreover, the non-overlapping expression of axonal marker phosphorylated Neurofilament Heavy Chain (p-NF-H) and dendritic marker MAP2, previously reported [64] in SH-SY5Y cells cultured and differentiated under the same conditions as we used here, also hinted at a mature phenotype. However, we cannot exclude the possibility that the SH-SY5Y cells did not secrete the proper myelin-promoting factors. Possibly, primary rodent neuronal cells or human iPSC-derived neuronal cells may therefore provide a more attractive substrate to study the myelinating potential of HOG and MO3.13 cells, but inclusion of primary or iPSC-derived neuronal cells was beyond the scope of our attempts to develop a high-throughput OL-neuron interaction model based on human cell lines.

A number of specific cell-biological processes that are operational in primary OLs and not or reduced in OL-related cell lines may be linked to the difficulty to differentiate the HOG and MO3.13 cells into myOLs. First, histone acetylation and fatty acid synthesis, two processes important for OL differentiation [88,89], may be affected in HOG cells because these cells lack N-acetylaspartate metabolism which is required for these processes [90]. Second, the difficulty to differentiate HOG and MO3.13 cells into myOLs could be due to the use of differentiation media that are deprived of glutamine in order to reduce cell proliferation. Glutamine starvation influences the Wnt/β-catenin pathway in oligodendroglial cells [91], which presumably affects OL myelination processes [92]. Furthermore, the level of expression of the glutamate-to-glutamine converting enzyme glutamine synthetase (GS) is low in undifferentiated HOG cells [91] and decreased in differentiated MO3.13 cells [36]. Third, MO3.13 cells have been found to express the OL-specific miRNA-338, but not the cooperative OL-specific miRNA-219 [93], which could directly affect OL differentiation [94,95]. Fourth, when compared to primary OLs the resistance of HOG cells to complement [96], and deviant responses of HOG and MO3.13 cells to extracellular stressors such as lipopolysaccharide (LPS) and tumor necrosis factor ligand superfamily member TRAIL [97,98] indicate that cell-biological processes other than myelin-related events occurring in cell lines cannot be directly compared to processes in primary OLs. Possibly, HOG and MO3.13 cells show such atypical (stress) responses because of their cancer-cell origin, since the HOG cell line is derived from oligodendroglioma and MO3.13 is constructed by fusion with a cancer cell line [5,6].

Surprisingly, in our experiments undifferentiated and differentiated HOG and MO3.13 cells showed expression of markers for non-OL related cell types. In particular, the N2.2 differentiation protocol induced mRNA expression of mature Schwann cell markers EGR2 and MPZ in HOG cells. A similar situation holds for the MO3.13 cells in which PMA, T3 and N2.2 differentiation caused an upregulation of EGR2 and/or the immature Schwann cell marker BLBP. The expression of peripheral glia markers in HOG and MO3.13 cells is in line with recent studies that show an overlap in marker expression between peripheral and central myelinating cells to a larger extent than previously thought [99]. The presence and modulation of neuronal (such as NSE and TUBB3) and astrocyte (such as SLC1A3, FGFR3 and PON2) markers at both the mRNA and protein level in both undifferentiated and differentiated HOG as well as MO3.13 cells raises the question whether these cell lines are purely glial in nature or that they display a mixed phenotype. Due to the large variety of markers expressed and their modulation by various small molecules, we presume that part of the undifferentiated HOG and MO3.13 cells may be in an OPC/O2A astrocyte progenitor state, since such cells are capable of differentiating into OLs as well as (type-1 and type-2) astrocytes [100,101]. This notion is supported by studies reporting expression of the astrocyte-specific proteins glutamate aspartate transporter 1 (SLC1A3) and glutamate transporter 1 (GLT-1) in undifferentiated HOG cells [102]. Also, a major feature of an astrocytoma cell line is the expression of neuronal and OL-related proteins and lipids [66], in line with a ‘mixed’ molecular phenotype of the HOG and MO3.13 cell lines [103]. 

Based on our results regarding the influence of HOG and MO3.13 cell density on mRNA expression of the OL-related marker PLP1, discrepancies between gene expression studies, such as concerning MBP expression in differentiated HOG and MO3.13 cells [14,24], may be explained by the seeding density of the cells. Yet, we found no effect of HOG and MO3.13 cell density on two non-OL-related transcripts, which implies that this effect may be transcript dependent. Our data are supported by Varini et al. [102] who showed that protein expression of SLC1A3 and GLT-1 was modulated by HOG cell density. To avoid such issues, cell density should be reported and taken into account in future in vitro culture studies on HOG and MO3.13 cells.

Our study has several limitations. First, we acknowledge that some of the cell-type markers used in this study are not specific, such as VIM that is both a neural progenitor and reactive astrocyte marker [104] and a number of Schwann cell markers such as BLBP have been described to be expressed in astrocytes as well [105]. However, since we have used multiple and in most cases cell-type-specific markers, our data substantiate the conclusion that HOG and MO3.13 cells do not represent a pure OL-phenotype. Second, we and others have omitted glutamine from the HOG and MO3.13 differentiation medium, which could have affected Wnt/β-catenin signaling and thus OL differentiation, although the exact role of this signaling pathway is at present not clear [106]. Third, we have provided only a first impression of the myelin-attractive and -repulsive environment in our co-cultures, and more adhesive factors should be tested. Finally, one should realize that the duration of the time period that we and others used to differentiate the HOG and MO3.13 cells is relatively short when compared to the in vivo OL differentiation time period.

In conclusion, with this study we have provided an extensive molecular and cellular characterization of undifferentiated and differentiated HOG and MO3.13 cells. The differentiation protocols applied here induced OL-related gene expression in differentiated HOG cells, though the resulting phenotype might not be purely OL related, but rather a ‘mixed’ astrocytic, neural and OL phenotype often found in astrocytoma cell lines. Similarly, we conclude that the morphology of MO3.13 cells differentiated with PMA and N2.1 was strongly reminiscent of the morphology of type-1 and type-2 astrocytes, which are shown in [107]. This finding, together with our marker expression data for undifferentiated and differentiated MO3.13 cells, suggests that also these cells represent a ‘mixed’ phenotype. Thus, our data clearly indicate that (un)differentiated HOG and MO3.13 cells represent heterogeneous cell populations and that the undifferentiated cells have the potential to differentiate towards cell-types other than (im)OLs, but not into myOLs. Moreover, co-cultures of HOG and MO3.13 cells with the differentiated human neuronal cell-line SH-SY5Y do not represent a valid model to study (re)myelination processes. Despite the apparent advantages of employing cell lines compared to primary cells in culture, we propose to reconsider or at least carefully deliberate the use of the HOG and MO3.13 cell lines as a model to study the (dys)functioning of human myOLs and pathological processes involved in OL-linked diseases. 

## Figures and Tables

**Table 1:** Differentiation media used in HOG and MO3.13 cell culture experiments. AA: antibiotic-antimycotic. db-cAMP: N6,2′-O-Dibutyryladenosine 3′,5′-cyclic monophosphate. IBMX: 3-isobutyl-1-methylxanthine. PMA: phorbol 12-myristate 13-acetate.

Differentiation Medium	Components
N2.1 medium	Eagle’s Minimum Essential Medium (EMEM) 1% antibiotic-antimycotic (AA), 50 μg/mL apo-transferrin, 16 μg/mL putrescine, 0.5 μg/mL human insulin, 30 nM sodium selenite, 30 nM triiodothyronine (T3), 500 uM 3-isobutyl-1-methylxanthine (IBMX), 500 uM dibutyryl cAMP (db-cAMP)
N2.2 medium	EMEM 1% AA, 50 μg/mL apo-transferrin, 16 μg/mL putrescine, 0.5 μg/mL human insulin, 30 nM sodium selenite, 30 nM T3, 10 ng/mL D-biotin, 50 nM hydrocortisone, 4 μM progesterone
T3 medium	EMEM 1% AA, 30 nM T3
PMA medium	Gibco Dulbecco’s Modified Eagle Medium: Nutrient Mixture F-12 (DMEM/F12(1:1)) 1% AA, 100 nM phorbol 12-myristate 13-acetate (PMA)

## Supplementary Materials

Supplementary data are available online at https://www.mdpi.com/2073-4409/8/9/1096/s1.
