# Supplementary material for: Reappraisal of Human HOG and MO3.13 Cell Lines as a Model to Study Oligodendrocyte Functioning
Source: Cells. 2019 Sep 17;8(9):1096. doi: 10.3390/cells8091096 (PMC6769895; doi:10.3390/cells8091096)
Supplement: Supplementary file 1 [file cells-08-01096-s001.pdf]

# Supplementary figures and tables

De Kleijn et al. (2019)

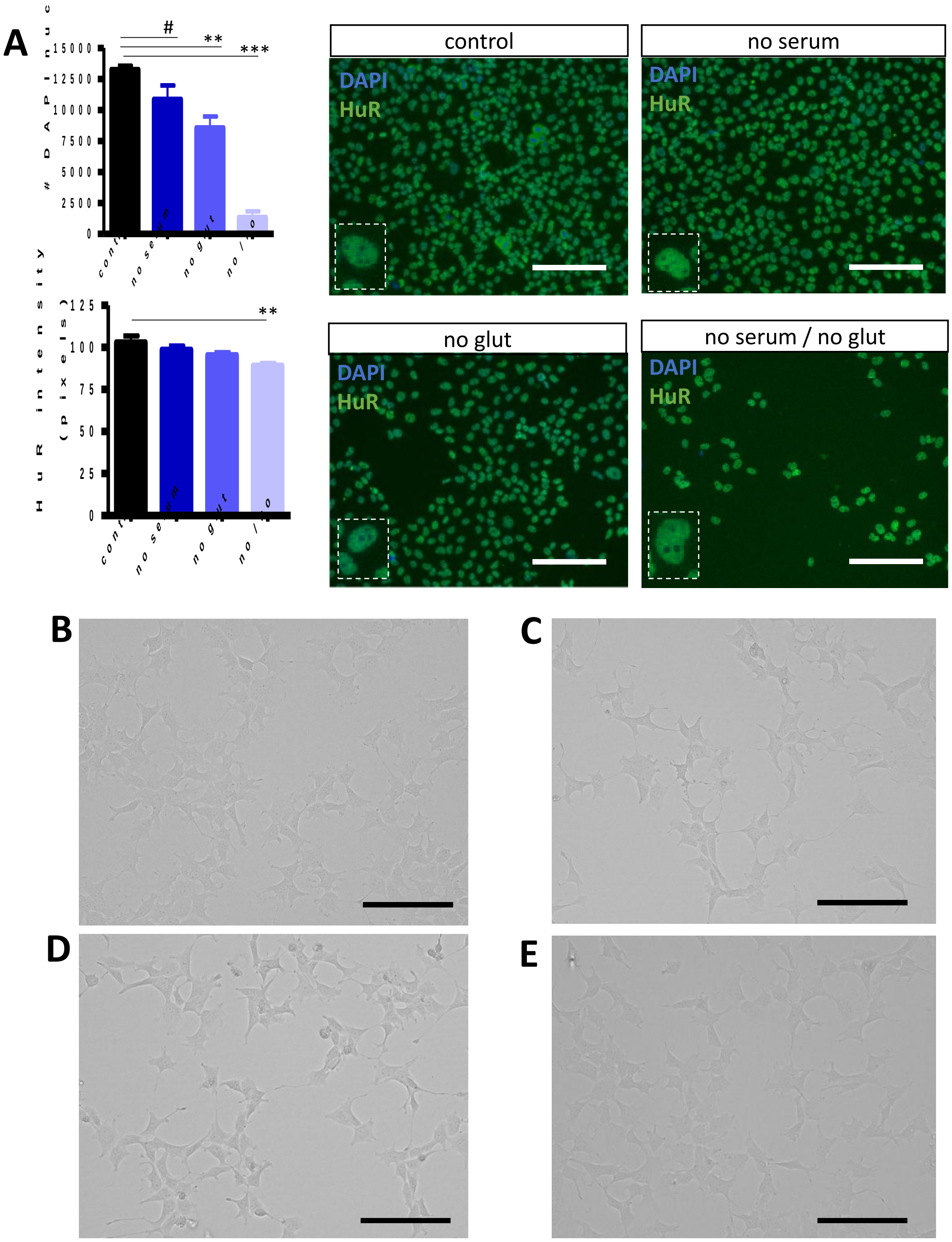


**Figure S1. (A)** HOG cell count (#DAPI nuclei), cytoplasmic HuR intensity quantifications and representative immunocytochemistry images of HOG cells cultured without serum, without Glutamax (glutamine) and without serum and Glutamax. Based on ANOVA with Tukey-b post-hoc comparisons on four independent experiments (n=4). P-values: * < 0.05, ** < 0.01, *** < 0.001. Morphology of **(B)** undifferentiated HOG and **(C)** HOG differentiated with N2.1 medium, **(D)** N2.2 medium and **(E)** T3. Scale bar = 50 μm.

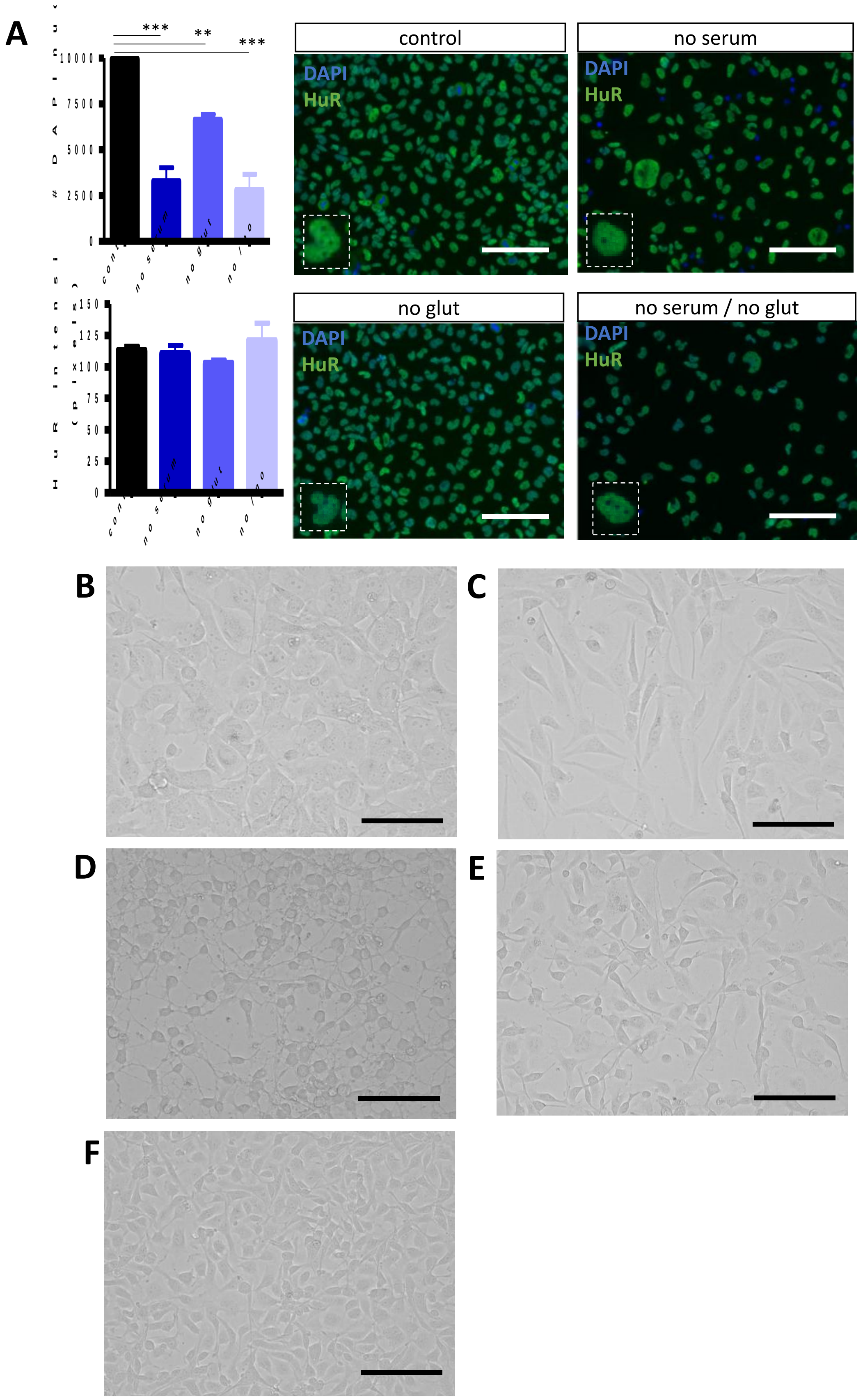


**Figure S2. (A)** MO3.13 cell count (#DAPI nuclei), cytoplasmic HuR intensity quantifications and representative immunocytochemistry images of MO3.13 cells cultured without serum, without Glutamax (glutamine) and without serum and Glutamax. Based on ANOVA with Tukey-b post-hoc comparisons on four independent experiments (n=4). P-values: * < 0.05, ** < 0.01, *** < 0.001. Morphology of **(B)** undifferentiated MO3.13 and differentiated with **(C)** PMA, **(D)** N2.1 medium, **(E)** N2.2 medium and **(F)** T3. Scale bar = 50 μm.

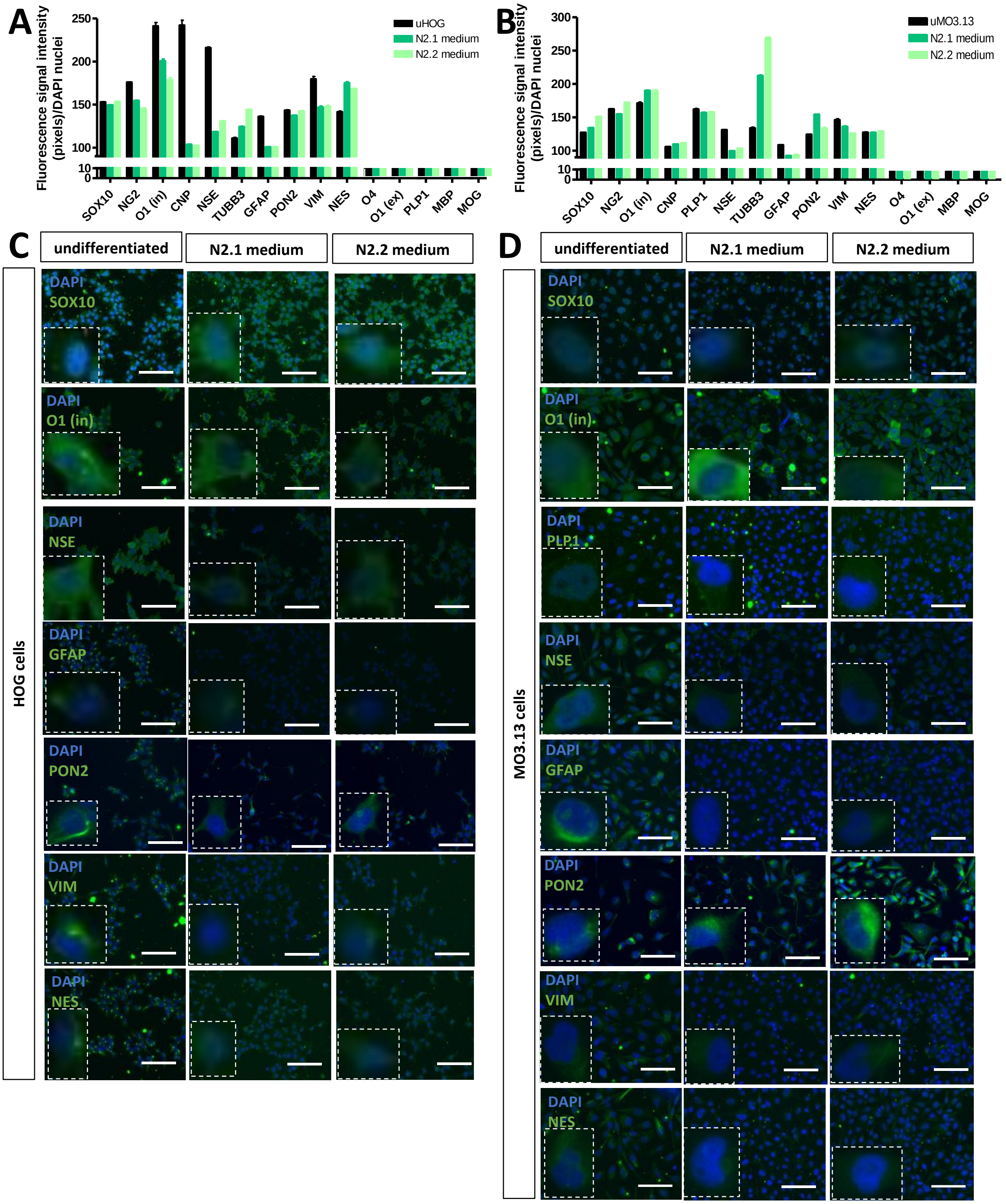


**Figure S3.** Immunocytochemical analyses in undifferentiated and differentiated HOG and MO3.13 cells. **(A)** Quantification of fluorescence signal in immunocytochemistry for undifferentiated HOG cells (uHOG) and HOG cells differentiated with N2.1 or N2.2 medium. Fluorescence quantification was based on one experiment (n=1), error bars = SEM of total number of DAPI-positive nuclei in one condition. **(B)** Quantification of fluorescence signal in immunocytochemistry for undifferentiated MO3.13 cells (uMO3.13) and MO3.13 cells differentiated with N2.1 or N2.2 medium. Fluorescence quantification was based on one experiment (n=1), error bars = SEM of total number of DAPI-positive nuclei in one condition. **(C)** Representative immunocytochemistry images for each marker in HOG cells and **(D)** MO3.13 cells. Scale bar = 50 μm.

**Table S1.** Variables and operationalisations used for SH-SY5Y and HOG or SH-SY5Y and MO3.13 co-culture experiments. OL: oligodendrocyte.

| Variable | Operationalisation |
|---|---|
| Coating | - Matrigel 1:100 (Corning)<br>- Poly-L-ornithine (Sigma) + Laminin (Sigma) |
| Total cell density | - $2.8 \cdot 10^5$ cells/cm$^2$<br>- $2.25 \cdot 10^5$ cells/cm$^2$<br>- $1.8 \cdot 10^5$ cells/cm$^2$<br>- $1.125 \cdot 10^5$ cells/cm$^2$<br>- $5.6 \cdot 10^4$ cells/cm$^2$<br>- $2.8 \cdot 10^4$ cells/cm$^2$ |
| OL to neuron ratio | - 1:1<br>- 1:2<br>- 1:3<br>- 1:6<br>- 1:9<br>- 1:18 |
| Co-culture duration | - 8 days<br>- Longer than 8 days |
| Pre-differentiation of OLs in mono-culture | - Yes<br>- No |
| Time-point of OL addition after neuronal differentiation initiation (day 10) | - Day 10<br>- Day 11<br>- Day 14<br>- Day 17<br>- Day 19<br>- Day21 |
| Medium change mode | - 100% of medium<br>- 50% of medium |
| Basal differentiation medium | - Neurobasal (Thermo Fisher)<br>- Brainphys (STEMCELL technologies) |
| Medium supplements | - D-Glucose (Merck)<br>- Sodium pyruvate (Gibco)<br>- Glutamax (Gibco)<br>- B27 supplement (Thermo Fisher)<br>- SM1 supplement (STEMCELL technologies)<br>- Culture-one supplement (Thermo Fisher) |
| Proteins/hormones | - Brain-derived neurotrophic factor (BDNF) (Sigma)<br>- All-trans retinoic acid (ATRA) (Sigma)<br>- Apo-transferrin (Sigma)<br>- Putrescine (Sigma)<br>- Triiodothyronine (T3) (Sigma)<br>- Sodium selenite (Sigma)<br>- Dibutyryl-cAMP (Sigma)<br>- Progesterone (Sigma)<br>- Human insulin (Sigma)<br>- Hydrocortisone (Sigma)<br>- D-biotin (Sigma)<br>- L-Glutamic Acid (Sigma)<br>- IBMX (Sigma)<br>- Ascorbic acid (Sigma)<br>- N-acetyl-cysteine (NAC) (Sigma)<br>- IGF-1 (Cell Guidance Systems) |

| | | |
|---|---|---|
| | - PMA (Sigma)<br>- Lithium chloride (LiCl)<br>- Sodium chloride (NaCl)<br>- Potassium chloride (KCl) | |
| Small molecules | - Miconazole (Sigma)<br>- Montelukast (Tebu-Bio)<br>- Clobetasol (Sigma)<br>- MDL29,951 (Tebu-Bio) | - Bradykinin (Sigma)<br>- Prosaptide (Sigma)<br>- Carbachol (Sigma)<br>- ATP (Sigma |

**Table S2.** Vendors and concentrations of antibodies used in immunohistochemistry (ICC) and Western blot (WB) experiments.

| Target | Vendor(clone) | Dilution for ICC | Dilution for WB |
|---|---|---|---|
| CC1 | Abcam (16794) | 1:500 | |
| CNPase | Aves Labs | 1:250 | 1:500 |
| DBH | Abcam (ab96615) | 1:500 | |
| FluoroMyelin Red | Thermo Fisher Scientific | 1:100 | |
| GAPDH | Cell Signaling Technology (14C10) | | 1:2000 |
| GAP43 | Aves Labs | 1:250 | |
| GFAP | UC Davis/NIH NeuroMab Facility (N206A/8) | 1:500 | |
| HuR | Santa Cruz (3A2) | 1:200 | |
| Jagged1 | Santa Cruz (E-12) | 1:500 | |
| L1CAM | Santa Cruz (5G3) | 1:500 | |
| MBP | Aves labs (polyclonal) | 1:500 | 1:2000 |
| MBP | Millipore (SMI99; monoclonal) | 1:500 | 1:500 |
| MOG | Home-made antibody CRICM, Pitié-Salpêtrière, Hospital, Paris | 1:50 | 1:1000 |
| MPZ | Aves Labs | 1:250 | |
| NEFL | Santa Cruz (DA2) | 1:500 | |
| NES | Millipore (MAB353) | 1:500 | |
| NG2 | Abcam (ab129051) | 1:100 | |
| NSE | Aves Labs | 1:250 | |
| O1 | Invitrogen | 1:250 | |
| O4 | Home-made antibody CRICM, Pitié-Salpêtrière, Hospital, Paris | 1:2 | |
| PLP1 | Abcam (ab28486) | 1:500 | 1:2000 |
| PON2 | Santa Cruz (C-5) | 1:500 | 1:250 |
| SOX10 | Abcam (EPR4007) | 1:100 | |
| TH | Millipore (AB152) | 1:500 | |
| TUBB3 | Biolegend (801201) | 1:1000 | 1:2000 |
| TUBB3 | Biolegend (802001) | 1:1000 | |
| VIM | DSHB (40E-C) | 1:50 | |

**Table S3.** Sequences of primers used in HOG and MO3.13 differentiation experiments. FW: forward primer, RV: reverse primer.

| Gene | Sequence | Gene | Sequence |
|---|---|---|---|
| BLBP FW | 5′-GAGACAAAGTGGTCATCAGGACTC | MYRF FW | 5′-CTTCAGCGTGGTGTCCATGTC |
| BLBP RV | 5′-CCATCCAGGCTAACAACAGACTTA | MYRF RV | 5′-GCAGCAAAGAGGGCTGTATGC |
| CNP FW | 5′-GGAGTACGCTCAACAAGATGTGA | NESFW | 5′-CGGGCTACTGAAAAGTTCCAG |
| CNP RV | 5′-CACAAAGAGGGCAGAGATGGT | NES RV | 5′-ACATCTTGAGGTGCGCCAG |
| DHH FW | 5′-GCCGTGCTTTGGACATCACTA | NEFL FW | 5′-GACCCTGGAAATCGAAGCATG |
| DHH RV | 5′-ATCAGCTTTGACCGACACGTG | NEFL RV | 5′-TTGATCGTGTCCTGCATAGCG |
| EGR2 FW | 5′-CACGTCGGTGACCATCTTTC | PDGFRa FW | 5′-GCCCGAGGAATGGAGTTTTT |
| EGR2 RV | 5′-ATCATGCCATCTCCGGC | PDGFRa RV | 5′-GCAGAAAGGTACTGCCTTTCGA |
| EIF4A2 FW | 5′-GGTGACATGGACCAGAAGGAGA | PLP FW | 5′-GCTGATGCCAGAATGTATGGTG |
| EIF4A2 RV | 5′-CCCCTCTGCCAATTCTGTGAA | PLP RV | 5′-CAATCATGAAGGTGAGCAGGG |
| FGFR3 FW | 5′- TCCTGCTCTGGGAGATCTTCAC | PPIA FW | 5′-CAGGGTTTATGTGTCAGGGTGG |
| FGFR3 RV | 5′- TGATCATGTACAGGTCGTGTGTG | PPIA RV | 5′- CCATTTGTGTTGGGTCCAGC |
| GAPDH FW | 5′-GTCATGGGTGTGAACCATGAGA | SLC1A3 FW | 5′- CTTCTTGGTAACACGGAAAAACC |
| GAPDH RV | 5′-GCATGGACTGTGGTCATGAGTC | SLC1A3 RV | 5′- TGGGTAGGGTGGCAGAACTT |
| GPR17 FW | 5′- GAGAGATGCTGAAACTCTCAGC | SOX10 FW | 5′-AGGAGAAGGAGGTTGACTGTTGC |
| GPR17 RV | 5′- CAGGGAGAAGTTGGTGATCAGAC | SOX10 RV | 5′-AGGTGCAGCCCCTCATCTTTC |
| GFAP FW | 5′-CCAGGACCTGCTCAATGTCAA | TUBB3 FW | 5′-GGGAAGTCATCAGTGATGAGCAT |
| GFAP RV | 5′-TCCAGGCTGGTTTCTCGAATC | TUBB3 RV | 5′-GAGGCACGTACTTGTGAGAAGAGG |
| MAG FW | 5′-CCAGGGAGCCCATCGAC | VCAN FW | 5′- GAATGTCACTCTAATCCCTGTCGT |
| MAG RV | 5′-GGTTGTCCCCTGCCGAG | VCAN RV | 5′- TCACATGTCTCGGTATCTTGCTC |
| MOBP FW | 5′-CCGTTCACCTTCCTCAATTCC | VEGF-A FW | 5′-GCTCAGAGCGGAGAAAGCATTT |
| MOBP RV | 5′-GCTGGTTCTGGTCTTCTGGC | VEGF-A RV | 5′-TCGGCTTGTCACATCTGCAAG |
| MOG FW | 5′-TTTTGATCCCCACTTTCTGAGG | VEGF-C FW | 5′-GGGCCAACCGAGAATTTGA |
| MOG RV | 5′-CGTAGCTCTTCAAGGAATTGCC | VEGF-C RV | 5′-GCCGTCTGTAACAGCTGCATGT |
| MBP FW | 5′-ACCCAAGATGAAAACCCCGTA | VIM FW | 5′-GAAATGGCTCGTCACCTTCGT |
| MBP RV | 5′-TCCGTAGCCAAATCCTGGTCT | VIM RV | 5′-GGAAGAGGCAGAGAAATCCTGC |
| MPZ FW | 5′-TGCAGAGGAGGCTCAGTGCTAT | YWHAZ FW | 5′-CGCTGGTGATGACAAGAAAGG |
| MPZ RV | 5′-CCTTGGCCTTCTTCTCACTGAC | YWHAZ RV | 5′-GAAGTTAAGGGCCAGACCCAGT |

**Table S4.** Differentiation studies in HOG and MO3.13 cells. ↓: a decreased expression relative to undifferentiated cells was reported. ↑: an increased expression relative to undifferentiated cells was reported. X: an absence of expression was reported. √: a presence of expression was reported.

| | HOG | | MO3.13 | |
|---|---|---|---|---|
| | **Undifferentiated** | **Differentiated** | **Undifferentiated** | **Differentiated** |
| **NG2** | | | | ↓ protein [25,34] |
| **PDGFRα** | | ↓ protein [25] | | ↓ protein [25,12] |
| **Gangliosides (A2B5)** | √ lipid [24] | √ lipid [24] | √ lipid [73] | |
| **Olig2** | | | √ protein [59] | ↑ mRNA [43]<br>↑ protein [43]<br>√ protein [59] |
| **CNPase** | √ mRNA [14]<br>√ protein [14]<br>X protein [24] | √ mRNA [14]<br>↑ protein [24] | √ mRNA [14]<br>√ protein [14,73,60]<br>X mRNA [35] | √ mRNA [14]<br>↑ mRNA [35]<br>↑ protein [34] |
| **GalC (O1)** | √ lipid [25,14]<br>X lipid [24] | ↑ lipid [24]<br>√ lipid [25] | √ lipid [14,25,73] | √ lipid [25] |
| **Sulfatide (O4)** | | | √ lipid [73] | |
| **CC1** | | | | √ ? [49] |
| **CGT** | | | | ↑ protein [12] |
| **FynKinase** | | ↑ mRNA [7] | | ↑ mRNA [7] |
| **OSP** | | | √ protein [60] | |
| **GST-p** | | | | ↑ protein [53,54] |
| **MBP** | √ protein [14]<br>X protein [24] | ↑ mRNA [14]<br>↑ protein [14]<br>X protein [24] | √ mRNA [14,35]<br>√ protein [14,73,37,59] | ↑ mRNA [14]<br>↑ mRNA [43,35]<br>↑ protein [27,34,73,60,43]<br>√ protein [37,40,38,59,47,50]<br>√ ? [49] |
| **PLP1** | X protein [18,21] | ↑ protein [21,18,20] | √ protein [60] | ↑ protein [34,12] |
| **OMG** | | ↑ mRNA [14] | | |
| **MOBP** | | ↑ mRNA [7,14] | | ↑ mRNA [7] |
| **MOG** | | ↑ mRNA [14] | | ↑ mRNA [14]<br>√ protein [40, 50] |
| **MAG** | | ↑ mRNA [14] | √ protein [60] | ↑ protein [34] |
| **VIM** | | | √ protein [73] | |
| **S100B** | | | | ↓ protein [34] |
| **GFAP** | X protein [14] | | X protein [14,59]<br>√ protein [37] | ↓ protein [34,37]<br>X protein [59] |

| | | | | |
|---|---|---|---|---|
| **PDGFRβ** | | | | √ protein [47] |
| **FGFR1** | | | | √ protein [47] |
| **FGFR3** | | | | √ protein [47] |
| **MAL2** | √ protein [21] | ↑ protein [21,20] | | |
